# The Effects of Dichloramine T on the Peritoneum

**Published:** 1918-08

**Authors:** 


**The Effet of Dichloramine T Chlorinated Eucalyptol Solution on the Peritoneum.** By S. P. Reimann and J. A. H. Magoun, *Surgery, Gynecology, and Obstetrics,* June 1918.

Reimann and Magoun report unfavorable results with this solution, which, when used as a 20 o/o solution, caused clotting of blood and exudate on gauze and drains, and led to interference with drainage and trauma. In dogs it produces a hemorrhagic fibrinous exudation. Even a 7 1/2 o/o solution on the dogs' peritoneum produces the same bad results and no benefits.

